# Generation of Monoclonal Antibodies Specific for Native LL37 and Citrullinated LL37 That Discriminate the Two LL37 Forms in the Skin and Circulation of Cutaneous/Systemic Lupus Erythematosus and Rheumatoid Arthritis Patients

**DOI:** 10.3390/antib9020014

**Published:** 2020-05-11

**Authors:** Roberto Lande, Raffaella Palazzo, Philippe Hammel, Immacolata Pietraforte, Isabelle Surbeck, Michel Gilliet, Carlo Chizzolini, Loredana Frasca

**Affiliations:** 1 Istituto Superiore di Sanità, National Center for Drug Research and Evaluation, Viale Regina Elena 299, 00161 Rome, Italy; roberto.lande@iss.it (R.L.); raffaella.palazzo@iss.it (R.P.); 2 Geneva Antibody Facility, Faculty of Medicine, University of Geneva, 1 rue Michel Servet, CH-1211 Geneva, Switzerland; philippe.hammel@unige.ch; 3 Istituto Superiore di Sanità, Department of Oncology and Molecular Medicine, Viale Regina Elena 299, 00161 Rome, Italy; immacolata.pietraforte@iss.it; 4 Department of Dermatology, University Hospital CHUV, 1011 Lausanne, Switzerland; isabelle.surbeck@chuv.ch (I.S.); michel.gilliet@chuv.ch (M.G.); 5 Department of Pathology and Immunology, Centre Médical Universitaire (CMU), Geneva University, 1 Rue Michel Servet, 1206 Geneva, Switzerland; carlo.chizzolini@unige.ch

**Keywords:** synthetic monoclonal antibodies, autoimmunity, protein post-translational modification (PTM), antimicrobial peptides

## Abstract

Human cathelicidin LL37 is a cationic antimicrobial peptide active against bacteria and viruses and exerting immune modulatory functions. LL37 can be also a target of autoreactive B- and T-lymphocytes in autoimmune settings. Irreversible post-translational modifications, such as citrullination and carbamylation, mainly occurring at the level of cationic amino acids arginine and lysine, can affect the inflammatory properties and reduce antibacterial effects. Moreover, these modifications could be implicated in the rupture of immune tolerance to LL37 in chronic conditions such as psoriatic disease and cutaneous lupus (LE)/systemic lupus erythematosus (SLE). Here, we describe the generation and fine specificity of six recombinant antibodies (MRB137–MRB142), produced as a monovalent mouse antibody with the antigen-binding scFv portion fused to a mouse IgG2a Fc, and their ability to recognize either native or citrullinated LL37 (cit-LL37) and not cross-react to carbamylated LL37. By using these antibodies, we detected native LL37 or cit-LL37 in SLE and rheumatoid arthritis (RA) sera, and in LE skin, by ELISA and immunohistochemistry, respectively. Such antibodies represent previously unavailable and useful tools to address relationships between the presence of post-translational modified LL37 and the immune system status (in terms of innate/adaptive responses activation) and the clinical characteristics of patients affected by chronic immune-mediated diseases or infectious diseases.

## 1. Introduction

Human cathelicidin is an antimicrobial peptide (AMP), also called CAP18 and FALL39, and is the product of the human gene CAMP. LL37, which corresponds to the COOH-terminal part of the molecule (res. 134–170), represents the mature form of human cathelicidin, released by epithelial cells and activated neutrophils, to figth infections by several bacteria, viruses and fungi [1,2,3,4,5]. LL37 is also present in neutrophil extracellular trap (NET)-like structures and is highly expressed in the skin, inflamed joints, and kidney in chronic diseases such as psoriasis, systemic sclerosis (SSc), psoriatic arthritis (PsA), rheumatoid arthritis (RA), and systemic lupus erythematosus (SLE) [6,7,8,9,10,11,12,13]. LL37 is also implicated in the activation of both the innate and adaptive immune system (IS) [6,10,14,15,16]. Being cationic, LL37 binds self and microbial DNA or RNA and activates important immune cells such as dendritic cells (DCs), monocytes and B-lymphocytes [6,8,17,18,19]. The capacity to form immune complexes with a particular structure renders LL37 a “danger” signal, able to stimulate TLR7, TLR8, and TLR9, crucial pathogen receptors involved in recognition of viral nuclei acids [20]. LL37 also becomes target of autoreactive B- and T-lymphocytes in autoimmune diseases such as systemic lupus erythematosus (SLE) and psoriatic disease (psoriasis, and PsA) [14,15,16]. It has been shown that LL37 is modified in vitro by citrullination and carbamylation, two types of irreversible post-translational modifications (PTMs), in which cationic amino acids are substituted by citrullines or homocitrullines, respectively [21,22]. These and other studies addressed the effect of such substitutions in vitro, showing impairment of some functions such as reduction of the antimicrobial activity, as well as gain of functions, such as increase of the immune-cell recruitment activity (chemotaxis) [21,22,23,24]. We recently identified anti-citrullinated LL37 (cit-LL37) and anti-carbamylated LL37 (carb-LL37) antibodies in the sera/plasma of SLE and PsA patients, respectively [15,16]. Citrullination and carbamylation are similar PTMs, because the substituted amino acids citrulline and homocitrulline are similar and both lower the cationicity of the molecule. Citrullination and carbamylation can be mediated by activation of protein arginine deiminases (PADs) and myeloperoxidase (MPO), respectively, both products of neutrophils among other cells, and indeed, both PTMs can be especially ascribed to neutrophil activity [25,26,27,28]. By using two of the monoclonal antibodies described here (MRB137 and MRB142), we have recently shown that native LL37 and cit-LL37 can be detected in tissues of SLE patients (kidney and skin) [14]. Here, we describe in more detail these antibodies, together with four additional monoclonal antibodies (raised in the same way, MRB138, MRB139, MRB140, MRB141), for their fine reactivity and capacity to discriminate not only between native LL37 and cit-LL37, but also between native or cit-LL37 and carb-LL37.

Such monoclonal antibodies represent previously unavailable tools to address the presence of mature human cathelicidin and its modified versions in body fluids such as sera/plasma and in tissues. The assessment of native and modified LL37 expression in tissues and circulation can help to clarify the characteristics and the outcomes of inflammation in infectious diseases, as well as in those chronic diseases in which the LL37 is up-regulated, as in psoriasis, as well as in other diseases such as RA, in which the citrullination of autoantigens appears to play a prominent pathogenic role [6,7,8,9,10,11,12].

## 2. Materials and Methods

### 2.1. Antigens Used in this Study

LL37: (LLGDFFRKSKEKIGKEFKRIVQRIKDFLRNLVPRTES), was purchased from Proteogenix (Schiltigheim, France).

cit-LL37: LLGDFFR(cit)KSKEKIGKEFKR(cit)IVQR(cit)IKDFLR(cit)NLVPR(cit)TES

Cit-REV: SETR(cit)PVLNR(cit)LFDKIR(cit)QVIR(cit)KEFEKGIKEKSKR(cit)FFDGLL

SCR: GLKLRFEFSKIKGEFLKTPEVRFRDIKLKDNRISVQR

Cit-SCR GLKLR(cit)FEFSKIKGEFLKTPEVR(cit)FR(cit)DIKLKDNR(cit)ISVQR(cit),

carb-LL37 (L*LGDFFRK*SK*EK*IGKEFK*RIVQRIK*DFLRNLVPRTES, in which the asterisks describe substitution with homocitrullines). To design this peptide, we referred to a previous study and tried to mimic high carbamylation of LL37 [22].

Carb-REV (SETRPVLNRLFDK*IRQVIRKEFEK*GIK*EK*SK*RFFDGLL*, in which the asterisks describe substitution with homocitrullines). These peptides were all synthesized by Citomatik (Italy). Peptides corresponding to the unrelated autoantigens vimentin, enolase and vinculin, in their native and citrullinated forms, were also used to further test the specificity and possible cross-reactivity of our monoclonal antibodies, as described [16].

### 2.2. Anti-LL37 Phage Display Selection Method

Briefly, a phage library displaying ScFv antibody fragments was deselected against Streptavidin coated Dynabeads (M280SA, Invitrogen 11205D) loaded at saturating conditions with a synthetic biotinylated scrambled LL37. The unbound antibody phages were then selected against beads loaded with a biotinylated LL37 peptide (Biot-LLGDFFRKSKEKIGKEFKRIVQRIKDFLRNLVPRTE-COOH). After 3 identical rounds of selection, the selected phages were analyzed by sequencing and expressed as mini-antibodies with the ScFv portion fused to mouse IgG2a Fc. By this method, we developed the anti-native LL37 antibodies.

To develop antibodies against cit-LL37, a phage library displaying ScFv antibody fragments was deselected against Streptavidin coated Dynabeads (M280SA, Invitrogen 11205D) loaded at saturating conditions with a biotinylated scrambled LL37. The unbound antibody phages were then deselected a second time against beads loaded with a biotinylated LL37 peptide. The unbound antibody phages were then selected against beads loaded with a biotinylated Citrullinated LL37 peptide, with 5 Arginins replaced by 5 Citrullines: (Biot-LLGDFF-Cit-KSKEKIGKEFK-Cit-IVQ-Cit-IKDFL-Cit-NLVP-Cit-TES–COOH). After 3 identical rounds of selection, the selected phages were analysed by sequencing and expressed as mini antibodies with the ScFv portion fused to mouse IgG2a Fc. By this method, we developed the anti-citrullinated LL37 antibodies.

### 2.3. ELISA for Detecting Antibody Specificity Using Peptide Antigens

The whole procedure was carried out at room temperature, as described [16]. LL37, cit-LL37 and SCR-LL37 were biotinylated and plated at saturating concentration (10 pmol/well) and immobilized on streptavidin-coated ELISA plates (Pierce #15124) for 30 min. Each well was rinsed three times with 100 μL of washing buffer (Phosphate-Buffered Saline (PBS) + 0.5% (*w*/*v*) Bovine Serum Albumin (BSA) + 0.05% (*w*/*v*) Tween20), then incubated for 1 h with 50 µL of each MRB antibody-containing supernatant diluted in washing buffer. After rinsing 3 times (100 µL washing buffer), wells were incubated with horseradish peroxidase-coupled (HRP) goat anti-mouse IgG (Bio-Rad #170-6516, dilution 1:1000, 50 μL per well) for 30 min. After 3 rinses, tetramethylbenzidine (TMB) substrate (Sigma #T5569) was added (50 μL per well). The reaction was stopped by the addition of 25 μL of 2 M H_2_SO_4_. The absorbance (OD) was measured at 450 nm, and the absorbance at 570 nm was subtracted.

For comparing reactivity of the antibodies not only to cit-LL37 but also to carb-LL37, non-biotinylated native LL37, cit-LL37 and carb-LL37, as well as cit-REV and carb-REV were directly coated on ELISA plates (Costar) and the procedure was carried out as above.

### 2.4. Human Studies

All sample sera and skin biopsies were obtained upon approval by Ethic Committees of CHUV/UNIL PROT. 335/12. Blood and tissue donors gave informed consent, according to Helsinki declaration. SLE disease activity was expressed as systemic lupus erythematosus disease index (SLEDAI, SLEDAI-2000) as described previously [16]. RA were diagnosed according to 2010 ACR/EULAR classification criteria as in previous studies [16].

### 2.5. ELISA for Detection of Native LL37 and cit-LL37 in Human Blood

Elisa plates were coated with MRB142 and MRB137 in ELISA plates (Costar) at a dilution of 1:50 in PBS 1% BSA. After 2 h at room temperature, plates were washed three times with washing buffer (PBS 1X + 0.5% BSA, 0.01% Tween20). Plates were saturated for 1 h with PBS 1X 4% BSA. After further 3 washes, 200 mL of diluted sera per well (usually 1:100 or 1:200 in PBS 1X, 1% BSA) were plated in duplicates and reaction lasted 2 h. After further three washes as above, we added the rabbit polyclonal IgG-antibody to LL37 (by Innovagen, Sweden), diluted 1:500 in PBS 1% BSA (200 µL per well). After further one-hour incubation plates were again washed as above. Then, we added the anti-rabbit IgG (Sigma), diluted 1:5000 in PBS 1%BSA (200 µL per well), conjugated with the horseradish peroxidase. After 1 h of incubation, plates were washed as above three times and TMB substrate was added (100 µL per well). The reaction was stopped by the addition of 50 μL of 2 M H_2_SO_4_. The absorbance (OD) was measured at 450 nm, and the absorbance at 570 nm was subtracted. Since this ELISA test is not quantitative, we used as references the sera from healthy donors (HD), representing the baseline. Sera were considered positive if the OD values obtained after testing the single patient serum sample was above the mean plus 3 standard deviations the OD values obtained in the group of the HD.

### 2.6. Detection of IFN-a in SLE Blood

IFN-α in sera was detected by using the ELISA kits for detection of human IFN-α (MabTech, Sweden). Sera were diluted 1:4 in PBS 1X 2% of BSA and added to the ELISA plates in 200 µL per well. ELISA was carried on according to manufacturer’s instruction.

### 2.7. Immunohistochemistry (IHC)

Staining was performed on 6-µm paraffin or frozen sections of human lupus erythematosus skin specimens obtained from CHUV, Dept. of Dermatology. Specimens were stained with the anti-LL37 (Innovagen, dilution 1:200, 5 µg/mL), followed by addition of anti-rabbit IgG (Vector Laboratories). Anti-mouse antibody and isotype control antibody (Vector) were also used. HRP and DAB (DAKO) were used to develop the color. Nuclear staining was performed with Mayer’s haematoxylin. Staining for native LL37 and cit-LL37 was performed with MRB138 and MRB139, on the same biopsies using the antibodies. MRB were used at 1:40 dilution, followed by incubation with a mouse antibody HRP (Abcam).

### 2.8. Statistical Analyses

Differences between mean values were assessed by Student’s t-test for single comparison (paired or unpaired samples, two-tailed). For patients’ study and for small N, we used a Wilcoxon matched-pairs signed rank test. We used Mann–Whitney test for a lower number of patients’ samples, for comparison between patients and HD. Statistical significance was set at *p* < 0.05. Correlation analyses were performed by Spearman’s rank-correlation test.

## 3. Results

### 3.1. MRB137 and MRB138 Recognize Exclusively Native LL37

**Figure 1:**
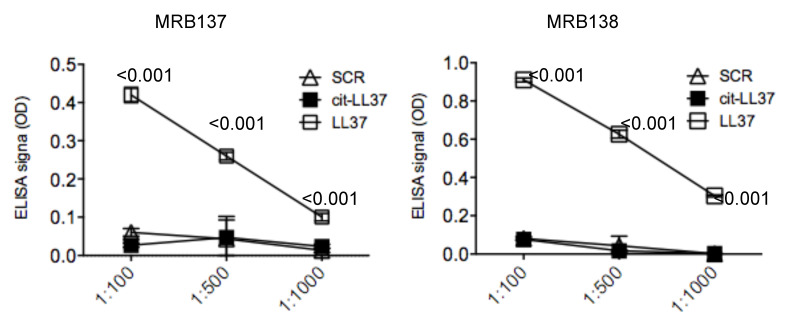
MRB137 and MRB138 recognize specifically native LL37. MRB137 and MRB138 (as specified in figure) were diluted in streptavidin ELISA plates coated with biotinylated native LL37 or cit-LL37 or control SCR LL37 peptide at the indicated dilutions, and ELISA tests were performed as in Material and Methods. Results are expressed as the mean of triplicate cultures. Standard errors or the mean are indicated. Representative results form three independent experiments. *p* values by paired Student’s *t*-test (two-tailed) refer to difference between recognition of native LL37 as compared to cit-LL37 or SCR LL37 peptides.

Antibodies MRB137 and MRB138, generated as described in Material and Methods, bound in a concentration-dependent manner to the native LL37 peptide, (against which they were raised), but not to the negative control scrambled peptide (SCR), nor to the cit-LL37 (Figure 1), in ELISA tests.

**Figure 2:**
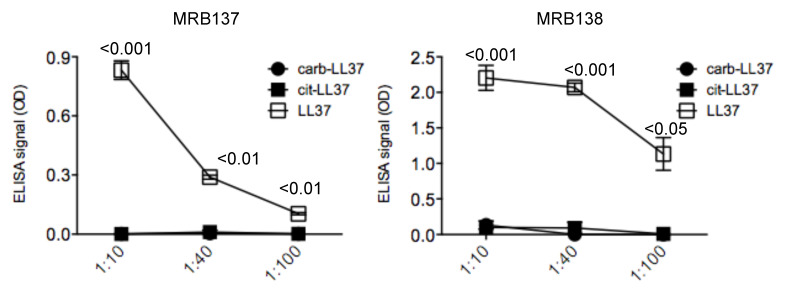
MRB137 and MRB138, specific for native LL37, do not cross-recognize carb-LL37. MRB137 and MRB138 were diluted in ELISA plates coated with native LL37, cit-LL37 or carb-LL37 peptide at the indicated dilutions and ELISA tests were performed as in Materials and Methods. Results are expressed as the mean of triplicate cultures. Standard error of the mean reported. Representative results form three independent experiments. *p* values by paired Student’s *t*-test (two-tailed) refer to difference between recognition of native LL37 and carb-LL37.

Moreover, in a subsequent test, we coated ELISA plates with native LL37, cit-LL37 and carb-LL37 and we confirmed the specificity of both antibodies MRB137 and MRB138 against the native LL37, whereas recognition not only of cit-LL37 but also of carb-LL37 was further excluded (Figure 2).

These results suggest that both antibodies could be used to address the presence of the native LL37 form in human body fluids and tissues.

### 3.2. MRB139, MRB141 and MRB142 Recognize Exclusively cit-LL37 and Do Not Recognize carb-LL37, Nor Citrullines or Homocitrullines Themselves

**Figure 3:**
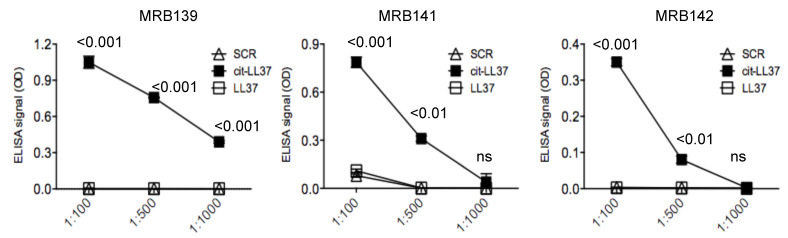
MRB139, MRB141 and MRB142 are specific for cit-LL37. MRB139, MRB141 and MRB142 were diluted in streptavidin ELISA plates coated with biotinylated native LL37 or cit-LL37 or control SCR LL37 peptide at the indicated dilutions and ELISA tests were performed as in Material and Methods. Results are expressed as the mean of triplicate cultures. Standard errors of the mean are indicated. Representative results form three independent experiments. *p* values by Student’s paired *t*-test (two-tailed) refer to difference between recognition of cit-LL37 as compared to native LL37 or SCR LL37 peptides.

Antibodies MRB139, MRB141 and MRB142 bound in a concentration-dependent manner to the cit-LL37 against which they were raised, but not to the negative control SCR peptide in ELISA tests, as represented in Figure 3.

**Figure 4:**
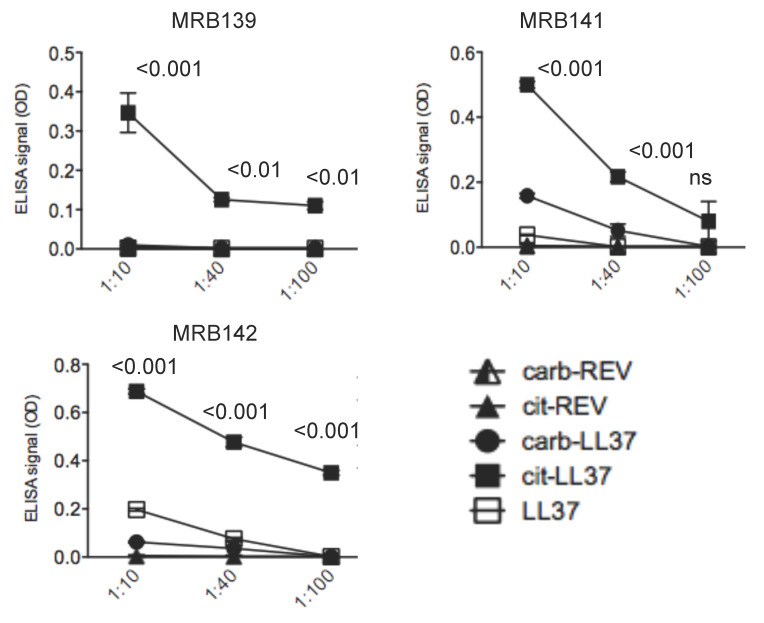
MRB139, MRB141 and MRB142 do not recognize carb-LL37 and are not merely citrulline or homocitrulline specific. MRB139, MRB141 and MRB142 were diluted in ELISA plates coated with native LL37 or cit-LL37 or carb-LL37 and control REV LL37 peptides (REV) either in their citrullinated or carbamylated form (cit-REV, carb-REV), at the indicated dilutions. ELISA tests were performed as in Material and Methods. Results are expressed as the mean of triplicate cultures. Standard errors of the mean are indicated. Representative results form three independent experiments. *p* values by Student’s paired *t*-test (two-tailed) refer to difference between recognition of cit-LL37 as compared to all the other control peptides as indicated.

Even in this case, we performed further assays after coating ELISA plates with native LL37, cit-LL37, and carb-LL37 to confirm the specificity of all three antibodies for cit-LL37, and exclude cross-recognition of carb-LL37 for all antibodies (Figure 4). As an important concern was the possibility that immune reactivity of MRB139, MRB141, and MRB142 could be due to reactivity to citrullines themselves and not to the sequence of LL37 as modified by citrullination. We have previously shown that this was not the case for MRB142 (Mab142 in [16]). Indeed, in a very recent work, we demonstrated that MRB142 recognizes cit-LL37 exclusively, as it did not recognize a LL37 reverse (REV) citrullinated peptide; moreover, MRB142 did not cross-react to other sequence unrelated molecules either, such as vimentin and enolase in their citrullinated forms [16]. Here, we performed similar experiments by using as control the REV LL37 peptide in its citrullinated form. As control, we also analyzed reactivity against a REV LL37 peptide in its fully carbamylated form. The results in Figure 4 showed again that specificity was towards cit-LL37 and not towards citrullines, whereas we also excluded cross-reaction of homocitrullines. These results suggest that the antibodies specific for the citrullinated form of LL37 truly recognize cit-LL37 and not the single amino acid citrullines. So, we can reasonable exclude that our antibodies represent citrulline-specific antibodies.

These results suggest all three antibodies could be used to address the presence of the cit-LL37 form in body fluids and tissues, without confounding cit-LL37 with carb-LL37.

### 3.3. MRB140 Recognizes Both Native and cit-LL37 But Not carb-LL37

**Figure 5:**
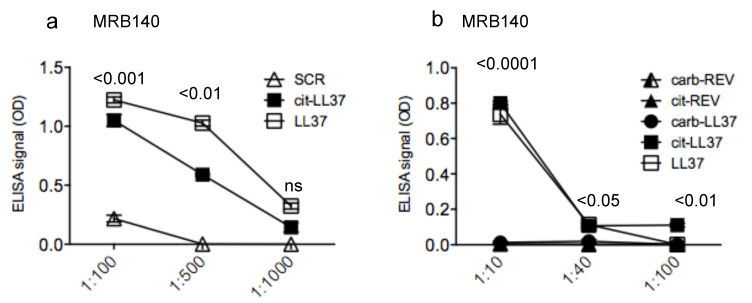
MRB140 is specific for both native LL37 and cit-LL37 but does not recognize carb-LL37 nor merely citrullines or homocitrullines. (**a**) MRB140 was diluted in streptavidin ELISA plates coated with biotinylated native LL37 or cit-LL37 or control SCR LL37 peptide at the indicated dilutions, and ELISA tests were performed as in Material and methods. (**b**) MRB140 does not recognize carb-LL37 nor citrulline and homocitrulline themselves. MRB140 was diluted in ELISA plates coated with native LL37 or cit-LL37 or carb-LL37 peptide or with control carb-REV or cit-REV peptides. ELISA tests were performed as in Material and Methods. In both (**a**) and (**b**) results are expressed as the mean of triplicate cultures. Standard errors of the mean are indicated. Representative results form three independent experiments. *p* values by Student’s paired *t*-test (two-tailed) refer to difference between recognition of cit-LL37 as compared to all the other control peptides as indicated.

Antibody MRB140 bound in a concentration-dependent manner to the cit-LL37, but also cross-recognized native LL37. This was unexpected, because MRB140 was raised against cit-LL37. The reactivity to native LL37 also tended to be slightly higher than that obtained in response to cit-LL37 (Figure 5a). Given the cross-reactive nature of MRB140, it was even more interesting to address reactivity to carb-LL37, to understand whether the recognition of both native LL37 and cit-LL37 was due to a degenerate recognition (Figure 5b). Interestingly though, MRB140 did not recognize the carb-LL37. Moreover, as the MRB139, 141, and 142, MRB140 did not recognize carb-REV or cit-REV (Figure 5b).

These results suggest that MRB140 could be used to differentiate cit-LL37 and carb-LL37, and the antibody is not merely directed towards citrullines or homocitrullines. Moreover, the results may also suggest that there is a limited overlapping between recognition of a monoclonal antibody of a citrullinated and a carbamylated LL37, likely due to differences in structures obtained with the two PTMs.

### 3.4. MRB137, MRB138 MRB139, MRB140, MRB141 and MRB142 Do Not Cross-Recognize Unrelated Autoantigens in Their Native or Citrullinated Form

**Figure 6:**
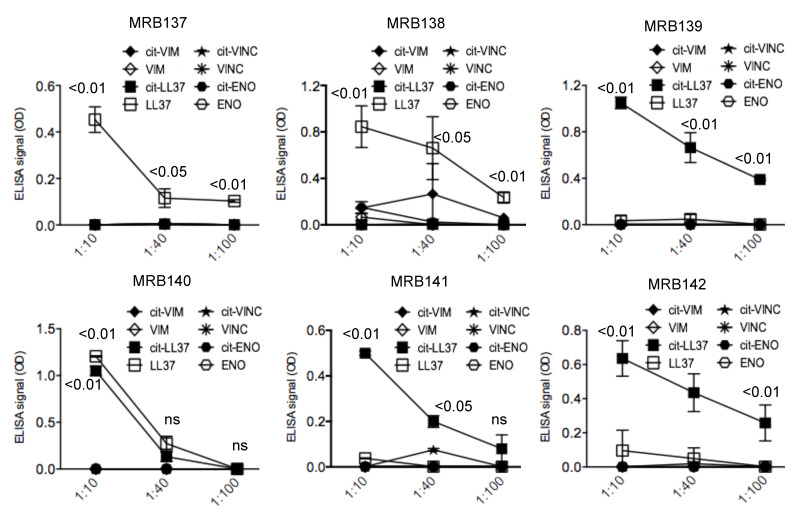
MRB137, MRB138, MRB139, MRB140, MRB141, MRB142 do not cross-react to unrelated peptides derived from other proteins acting as autoantigens in autoimmune diseases. All monoclonal antibodies were tested in ELISA tests as above, to peptides derived from vimentin (VIM), enolase (ENO), vinculin (VINC), in their native or citrullinated (cit-) forms, as indicated. Results are expressed as the mean of three different experiments. Standard errors of the mean are indicated. *p* values by paired Student’s *t*-test (two-tailed) refer to difference between recognition of native LL37/cit-LL37 as compared to all the other unrelated autoantigens; ns: not significant.

The capacity of the monoclonal antibodies tested above to recognize specifically native LL37 or cit-LL37 can be useful for detection of the two forms in ex vivo tissues or body fluids. Other autoantigens have been described to be citrullinated in some autoimmune settings, or in chronic inflammation. Some of these proteins are ubiquitously expressed such as vimentin, enolase, as well as vinculin [16,29,30,31]. To more finely study the cross-reactivity of the antibodies we tested all the antibodies against peptides derived from these proteins and we found no cross-reactivity in ELISA tests, performed as in the previous experiments (Figure 6). Thus, these further pieces of data regarding typical autoantigens recognized in more than one autoimmune disease further indicate the specificity of our antibodies.

### 3.5. MRB138 and MRB139 Discriminate Expression of Native and cit-LL37 in Human LE Skin

**Figure 7:**
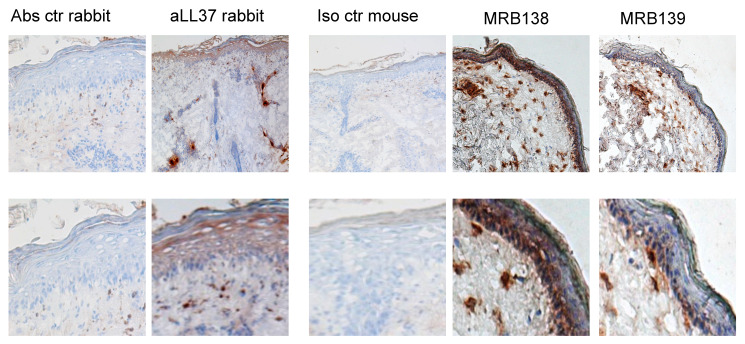
MRB138 and MRB139 recognize native LL37 and cit-LL37 in different locations in SLE skin. Frozen skin of LE patients was stained with MRB138 or MRB139 as described in Materials and methods. As reference, skin was also stained with polyclonal rabbit anti-LL37 (Innovagen) (See also Figure 7) as indicated. Control antibody staining was also performed. Representative experiments of two performed. Magnification: 20×, insets in the lower panels.

**Figure 8:**
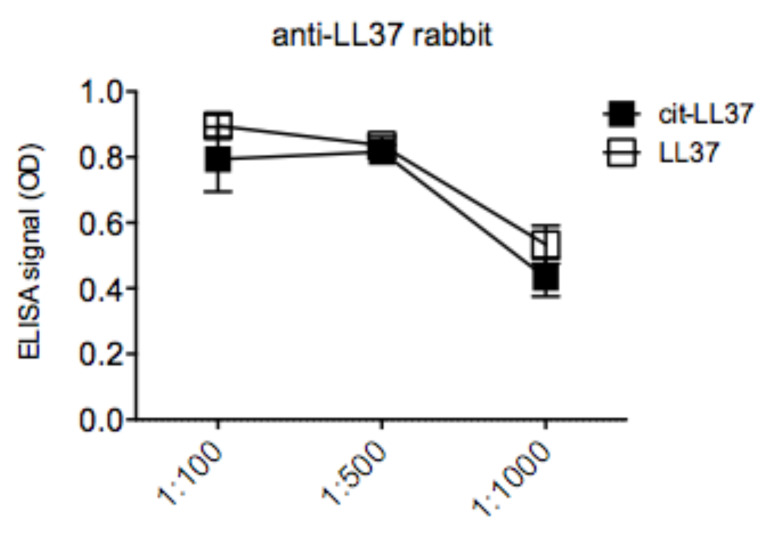
Polyclonal rabbit anti-LL37 does not discriminate between LL37 and cit-LL37 in ELISA tests. The rabbit polyclonal anti-LL37 (Innovagen) was diluted, as indicated, in ELISA plates coated with native LL37 or cit-LL37 peptides. Results are expressed as the mean of triplicate cultures. Standard errors of the means are indicated. Representative results form two independent experiments.

We have recently demonstrated expression in lupus erythematosus (LE) skin of both native LL37 and cit-LL37, by IHC [16]. It was already known that LE and SLE skin express high level of LL37 as compared to healthy skin [16,32,33]. However, the presence of cit-LL37 had never been assessed in those studies. In our recent paper, we showed that MRB142 was able to reveal the presence of consistent amounts of cit-LL37 in LE skin, whereas MRB137 detected the native LL37 [16]. The distribution of native LL37 and cit-LL37 in tissues from the same patients was similar, in that at least 40–50% of LL37 appeared citrullinated in LE skin. The same was shown in SLE-affected kidney [16]. Here, we used the two additional monoclonal antibodies MRB138 (specific for native LL37) and MRB139 (specific for cit-LL37), to detect ether native LL37 or cit-LL37 in LE skin. The results obtained as depicted in Figure 7, are in keeping with previous results obtained with MRB142 and MRB137 (the latter being the other monoclonal antibody recognizing exclusively native LL37) [16]. It is worth to notice that the staining indicates that cit-LL37 is mainly detected in the dermal compartment, and much less in the epidermis. In contrast, the staining with the antibody specific for native LL37 (MRB138) also detects LL37 in the subtle epidermal stratum of LE biopsies. As a reference, we used the commercially available rabbit anti-LL37 polyclonal antibody preparation by Innovagen (see Methods), which also stains both the dermis and epidermis of LE skin [14,16]. Of note, we demonstrate in ELISA tests, that the rabbit anti-LL37 antibody preparation is able to recognize LL37 in both its native and citrullinated form (Figure 8). Since LE dermis is usually characterized by a neutrophil infiltrate, the results obtained are in line with the assumption that LL37 produced by keratinocytes in epidermis is mainly maintained in its native form, whereas cit-LL37 detected in the dermis could be the result of neutrophilic inflammation and neutrophil signature, which could favor citrullination phenomena [28,34,35].

### 3.6. Mab137 and MRB142 Can Be Used in ELISA Tests to Assess LL37 and cit-LL37 Expression in Body Fluids

**Figure 9:**
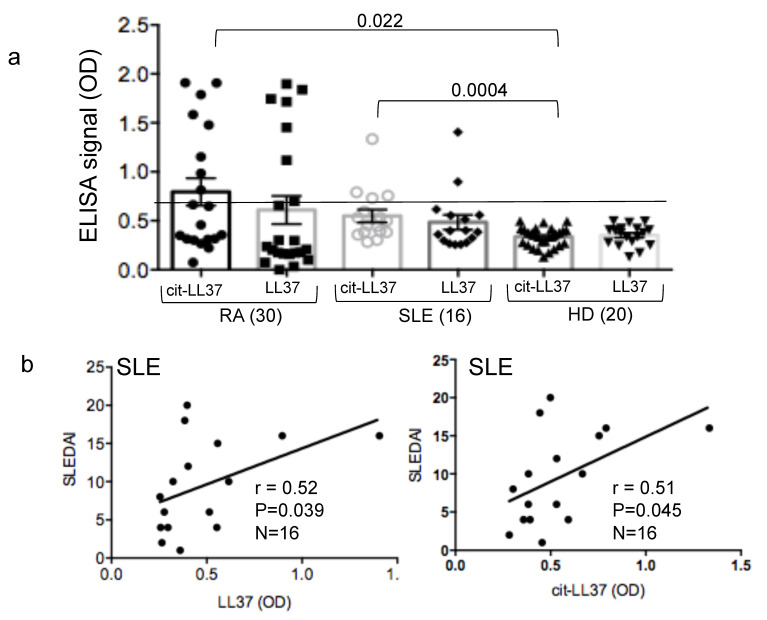
Native LL37 and cit-LL37 are present in circulation of RA and SLE patients. (**a**) RA and SLE sera were diluted 1–100 in ELISA plates coated with MRB139 or MRB142 (1:50) and ELISA was performer as in Material and Methods. Horizontal bars are the means; vertical bars are standard errors of the mean, *p* values calculated by Mann-Whitney test. Sample size indicated in brackets. The horizontal line indicates cut-off determined by reactivity of HD, as explained in Material and Methods. (**b**) Correlation by Spearman’s correlation test (two-tailed) between presence of native LL37 or cit-LL37 in blood and disease status (expressed as clinical SLEDAI).

Next, we explored whether the antibodies described above could be used to set-up an assay for the detection of LL37 or cit-LL37 peptides in body fluids of patients in which LL37 is suspected to play a role in inflammation. For instance, it has been shown that LL37 can be up-regulated in tissues and in circulation of SLE patients, and some up-regulation of LL37 has been also seen in joints as well as in the circulation of RA patients [11,12,13,16,32,33]. Therefore, we have set-up an ELISA test, in which we have coated plates either with the anti-native LL37 antibody (MRB137) or the anti-cit-LL37 antibody (MRB142) to capture LL37 or cit-LL37 directly from diluted patients’ sera, and used the commercial rabbit anti-LL37 antibody preparation mentioned above (which detect both native and cit-LL37, as in Figure 7) to reveal the binding of either native LL37 or cit-LL37 to the plate coated with these two antibody. We have analyzed SLE and RA sera and, since the assay was not quantitative, we used a reference control a group of sera from healthy donors (HD). HD sera served as the baselines and we considered positive the sera as described in Section 2. Our data showed circulating levels of both native LL37 and cit-LL37 in some SLE and RA patients, which was expected from the literature (Figure 9a). Indeed, eight and six RA patients out of 30 (27% and 20%, respectively) showed elevation of cit-LL37 or native LL37 compared to baseline, (HD). Moreover, four and two out of 16 SLE patients showed elevated levels of cit-LL37 or native LL37 in circulation (25% and 13%, respectively). Of note, elevation of cit-LL37, unlike elevation of native LL37, was significant in both SLE and RA patients. However in SLE, we find a positive and significant correlation between intensity of expression of both native LL37 and cit-LL37 and SLEDAI in patients as reported in Figure 9b. Interestingly, although only four SLE patients expressed detectable IFN-α levels in their blood, we found a positive and significant correlation between presence of native LL37 or cit-LL37 in sera and positivity for IFN-α (Spearman correlation between native LL37 and serum IFN-α: r = 0.47, P = 0.035, N = 16, one-tailed test; Spearman correlation between cit-LL37 and serum IFN-α (pg/mL): r = 0.52, P = 0.02, N = 16, one-tailed test). We could not find any relationship between the detection of cit-LL37 and native LL37 in RA sera and disease parameters such as ESR (erythrocyte sedimentation rate) and CRP (C-reactive protein) (not shown), in RA patients. These results suggest that the two monoclonal antibodies MRB139 and MRB142 can discriminate between presence of native LL37 and cit-LL37 in sera, respectively, although larger clinical studies are needed to confirm the importance of detection of native LL37/cit-LL37 in circulation in chronic diseases such as RA and SLE.

## 4. Discussion

In this paper, we report the successful generation of new monoclonal antibodies that are able to specifically recognize either native LL37 or cit-LL37. This is of interest in that LL37 is an AMP with important functions in the IS as it possesses interferogenic properties and is able to stimulate pro-inflammatory mediators [6,7,16,17,18,19,20,27,28]. Of note, LL37 can also limit inflammation during infections and sepsis, by binding and neutralizing bacterial lipopolysaccharides (LPS), an effect mediated by its cationic charged amino acids favoring binding to the polyanionic LPS [24,36].

Thus, the possibility to dispose of reliable antibody tools to assess presence in tissues and body fluids of native LL37 or cit-LL37 can allow monitor LL37 modification in infectious diseases, as well as in those immune-mediated diseases characterized by abnormal LL37 expression. Diseases characterized by aberrant LL37 expression include, apart from psoriasis, RA, in which LL37 and other AMPs have been found in synovial fluids and circulation (sites of neutrophilic inflammation), SLE, in which LL37 is present in affected organs such as the skin and kidneys, as well as systemic sclerosis (SSc), characterized by high LL37 expression in affected skin [6,7,8,9,10,11,12,13,14,15,16,32,33]. We have also demonstrated that LL37 acts as an antibody and/or T-cell auto-antigen in SLE, psoriasis, and PsA [14,15,16].

To date, our antibodies are unique in discriminating native LL37 from cit-LL37 and the demonstration that they do not recognize ubiquitously expressed autoantigens, such as vimentin and enolase, suggests their use in autoimmune diseases, particularly in RA [29,30,31].

Previous antibodies have been described to recognize only native LL37, whereas cit-LL37 was recognized by western blot only using an anti-citrulline antibody [23,24,25]. We have definitely excluded that reactivity to cit-LL37 of our antibodies is due to recognition of citrulline itself. Thus, these antibodies are likely conformation specific but also sequence specific. Moreover, lack of cross-reactivity with carb-LL37 is also an interesting feature. Indeed, carbamylation and citrullination are similar modifications and citrulline and homocitrulline differ very little [25]. At the same time, although both PTMs reduce the cationic charge of LL37, their effect on LL37 functions are not exactly the same. While antimicrobial activity is reduced by both PTMs, carbamylation increases the LL37-driven neutrophils chemotaxis, whereas citrullination decrease it [22,23,24]. Thus, the possibility to discriminate between cit-LL37 and carb-LL37 is a plus, because both citrullination and carbamylation are in some way linked to the same effector cells, namely to neutrophil activity. Indeed, citrullination is mediated by calcium activated PAD enzymes, which can be released by neutrophils during inflammation or become activated inside the cells by various pathways [27,32]. However, neutrophils are also linked to carbamylation because they release MPO when activated. It is indeed the MPO that catalyzes the formation of cyanate from hydrogen peroxide and thiocyanate, ultimately leading to carbamylation [26,27,28].

An interesting ability of the antibodies MRB138 and MRB139 is that they discriminate the skin location of LL37 by IHC. Native LL37 is present in both the epidermis (where it is produced by keratinocytes) and also in the dermis, as it is a marker of neutrophils, cells known to infiltrate inflamed lupus skin at a high level [34,35]. Instead MRB139, as well as MRB142 [16], mainly detect cit-LL37 in the dermal compartments, in line with the observation that cit-LL37 is predominantly produced and released where the neutrophil infiltrate is present.

These antibodies are certainly helpful tools for researchers. At present, studies in large cohorts of patients with RA or SLE or other autoimmune diseases, which address the correlation between patient subgroups and the presence/absence of native LL37 and cit-LL37 in target tissues, are not available. The same is true for studies that address the clearance of pathogens in relationship to modification of LL37 in tissues and circulation. The presence or absence of native LL37 or cit-LL37 could be correlated to particular inflammatory cytokines such IFN-α, TNF-α, IL-6 in clinical studies [22,24], but more observations are needed. By using the antibodies described here, such studies can be more simply made, because less work is required to address PTMs as compared to other ways, such as extractions of proteins followed by HPLC from tissues, and western blots using anti-citrulline antibodies, which are more challenging [25].

From the clinical point of view, once more studies are made, it is not excluded that these antibodies can become diagnostic tools. As a more immediate use, they could be exploited to study the general level of protein citrullination, in several conditions, using LL37 as a reference molecule, by IHC or ELISA (the latter on body fluids). We have not tested yet whether the antibodies also work to discriminate LL37 and cit-LL37 by western blot. This can be done using synthetic LL37 and cit-LL37 in these tests. However, discrimination in western blots from tissue extracts can be more difficult, in that the molecular weight of native LL37 and cit-LL37 differs very little, so the analysis can be less informative using these antibodies, and render more difficult the quantification as compared to ELISA tests. IHC has the prerogative to detect LL37 and cit-LL37 more precisely, as it discriminates their locations [16].

Finally, we want to add that the capacity of MRB140 to cross-recognize LL37 and cit-LL37 is interesting, especially because MRB140 was raised and selected for reactivity to cit-LL37 and not native LL37. So, the existence of a monoclonal antibody with such double specificity suggests that it is possible to select antibodies recognizing a self-antigen using a modified version of the self-antigen. Of course, the propagation of such antibody types in vivo, if present in patients with autoimmune diseases, could be subjected to regulation by T-cells [16]. However, we demonstrated in SLE that antibodies to native LL37 are induced in vitro also in the presence of anti-cit-LL37 reacting T-cells and do not necessarily need the activation of a T-cell specific for the native peptide [16]. Interestingly, the MRB140 does not recognize carb-LL37. This may be due to the production and selection methods as a recent paper has shown that monoclonal antibodies derived from RA patients, and reactive to citrullinated proteins, were very frequently able to recognize the same proteins bearing different PTMs such as carbamylation or acetylation [37].
